# Multiple Gastric Adenomyomas Mimicking Leiomyoma: A Novel Case Report

**DOI:** 10.1002/kjm2.70018

**Published:** 2025-03-31

**Authors:** Chen Yuan, You‐Hong Cao

**Affiliations:** ^1^ Department of Gastroenterology Gaochun People's Hospital of Nanjing Nanjing China

A 68‐year‐old female patient was referred to our institution following the incidental discovery of a 10‐mm submucosal protrusion along the greater curvature of the gastric antrum during routine endoscopy (Figure 1A). Contrast‐enhanced abdominal CT revealed nonspecific findings without definitive malignant characteristics such as significant lymphadenopathy or metastatic lesions. Subsequent endoscopic ultrasonography (EUS) demonstrated a well‐circumscribed hypoechoic mass originating from the superficial muscularis propria layer, radiologically suggestive of leiomyoma (Figure 1B). Elective endoscopic submucosal dissection (ESD) was performed for definitive histopathological evaluation. The procedure and postoperative recovery proceeded without complications, with successful discharge on postoperative Day 5 (Figure 1C,D). Histopathological analysis unexpectedly identified three distinct subcentimeter lesions (all < 10 mm) exhibiting characteristic adenomyomatous features—ectopic glandular structures embedded within hyperplastic smooth muscle stroma (Figure 1E,F).

**FIGURE 1:**
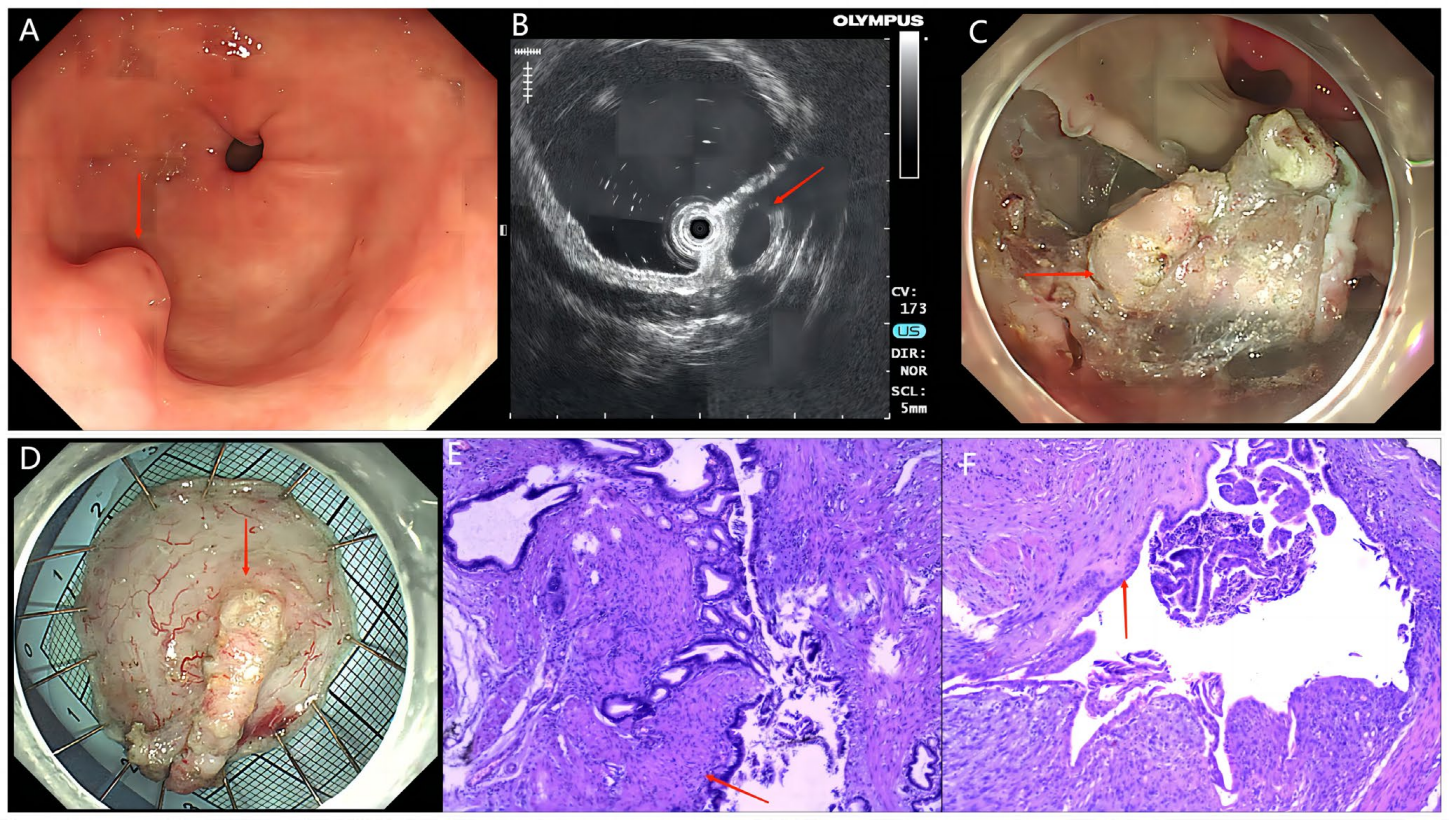
A 10 mm sized submucosal bulge along the greater curvature in the gastric antrum (A). Endoscopic ultrasonography presented heterogeneous echo (B). An ESD was on scheduled (C, D). Histopathological examination revealed the diagnosis of multiple gastric adenomyoma (E, F).

Gastric adenomyoma represents an exceptionally rare benign neoplasm characterized histologically by ectopic glandular structures embedded within hyperplastic smooth muscle stroma [1]. While classically described as solitary lesions predominantly located in the gastric antrum or pyloric region (> 85% of reported cases), this report describes the first documented instance of primary multifocal gastric adenomyomatosis, presenting three distinct lesions—a finding that fundamentally contradicts existing histopathological paradigms [1]. The tumor typically originates from the submucosal layer (67% of published cases), with less frequent involvement of the muscularis propria (33%). Although preoperative endoscopic ultrasonography (EUS) provides critical localization data (muscularis propria, PM), our case underscores its diagnostic limitations: The current lesion's superficial muscularis propria origin and homogeneous hypoechoic pattern mimicked conventional leiomyoma, necessitating definitive histopathological confirmation. This diagnostic challenge stems from adenomyoma's dual clinicopathological signature—concurrent proliferation of both glandular epithelium (CK7+/CK20+ immunophenotype) and smooth muscle components (α‐SMA+/desmin+) [2]. Notably, three novel clinicopathological observations emerge from this case: (1) Multifocality challenging current etiological models of isolated adenomyoma development.

(2) Muscularis propria origin contrasting with predominant submucosal localization patterns. (3) Subcentimeter dimensions (< 10 mm) complicating preoperative detection. While current literature suggests generally favorable prognosis, emerging evidence identifies malignant transformation potential through adenomyoma‐adenocarcinoma sequence progression (3.8% risk in longitudinal studies) [3]. In this patient, surveillance was prioritized given the absence of high‐risk features (lymphovascular invasion, nuclear atypia, or mitotic activity > 5/50 HPF). Long‐term endoscopic monitoring at 6‐month intervals has been instituted to assess potential lesion evolution.

## Conflicts of Interest

The authors declare no conflicts of interest.

## Data Availability

The data that supports the findings of this study are available in the Supporting Information of this article.
